# Inflammation preservation strategy: reconciling pain control and disc resorption in lumbar disc herniation

**DOI:** 10.3389/fimmu.2025.1653681

**Published:** 2025-09-02

**Authors:** Guanyi Gong, Zheng Yan, Qilong Lai, Peijie You, Pengfei Yu, Xiaochun Li, Zhiqiang Wang, Shun Lin, Yuxiang Dai, Hong Jiang, Jintao Liu

**Affiliations:** Suzhou TCM Hospital Affiliated to Nanjing University of Chinese Medicine, Suzhou, China

**Keywords:** lumbar disc herniation, inflammation preservation strategy, macrophage polarization, disc resorption, precision immunomodulation, spontaneous resorption, lumbar disc extrusion, inflammatory microenvironment modulation

## Abstract

Lumbar disc herniation (LDH) is a prevalent condition driven by inflammation, which mediates both radicular pain and spontaneous resorption of herniated material. Traditional anti-inflammatory therapies alleviate pain but may impede disc regression. We propose an Inflammation Preservation Strategy (IPS) to harness inflammation’s reparative potential while managing symptoms. Molecular, clinical, and translational evidence reveals inflammation drives resorption in 60–90% of LDH cases. Key mechanisms include neovascularization, dynamic macrophage polarization (where M1 degrades matrix while M2 promotes repair), and apoptosis-autophagy synergy. Traditional anti-inflammatory therapies risk suppressing this reparative cascade, whereas IPS advocates precision modulation—avoiding pan-anti-inflammatory agents during acute phases and employing targeted interventions to balance analgesia with tissue healing. Clinical data support IPS in achieving near-complete resorption and sustained pain relief, suggesting a paradigm shift from symptomatic palliation to disease-modifying regeneration. Future directions include real-time inflammation phenotyping and smart biomaterials to advance precision IPS implementation.

## Introduction

Lumbar disc herniation (LDH) represents a prevalent orthopedic condition, with documented annual incidence rates ranging from 1.6% in the general population to as high as 43% among certain occupational cohorts characterized by repetitive heavy lifting or spinal loading (1). Epidemiological analyses show LDH disproportionately contributes to global low back pain burden, accounting for 18-22% of chronic cases. It is a leading driver of disability, particularly among women aged 45–49 years. In this demographic, LDH-related years lived with disability (YLDs) peak at over 1,600 per 100,000 population, paralleling the highest rates reported in the Global Burden of Disease 2021 (2). Furthermore, since 1990, medium socio-demographic index regions have experienced a 12.9% rise in the age-standardized prevalence of LDH (3).

Inflammation serves as the central pathophysiological mechanism underlying LDH-associated pain, with inflammatory mediators acting as the primary driving force behind the transition from acute to chronic pain through sustained activation of nociceptive pathways and promotion of neural plasticity (4). Traditional therapeutic paradigms focus on suppressing acute inflammatory responses, based on the concept that cytokine storms (e.g., TNF-α, IL-1β, PGE_2_) directly stimulate nerve roots, triggering radicular pain (5, 6). International surveys indicate that non-steroidal anti-inflammatory drugs (NSAIDs) and glucocorticoids are widely adopted as first-line pharmacotherapies for LDH by spine surgeons (7). Cochrane reviews (8–10) emphasize NSAIDs as the only oral non-opioid agents with dual anti-inflammatory and analgesic effects, positioning them as primary interventions for low back pain. The North American Spine Society (NASS) guidelines (11) recommend NSAIDs as preferred acute-phase treatment for LDH.

However, emerging evidence (12–14) reveals inflammation as a key driver of herniated disc resorption. Spontaneous resorption occurs in 60%–90% of extruded and sequestrated LDH cases, with inflammation serving as the core mechanism. Fundamental studies (12, 15, 16) confirm this process relies on a cascade of neovascularization, macrophage infiltration, and matrix degradation. Non-surgical management promotes size reduction of herniations, particularly in extruded/sequestrated subtypes (17).

A critical paradox emerges: While inflammation is traditionally viewed as a pain inducer, new data highlight its essential role in spontaneous disc regression. Anti-inflammatory therapies, despite symptom relief, may impede resorption (18). Thus, we propose the Inflammation Preservation Strategy (IPS), advocating avoidance of potent anti-inflammatory agents during the acute phase to harness localized inflammation for herniation clearance.

Unlike traditional conservative management (e.g., NSAIDs, glucocorticoids) that globally suppress inflammation, or targeted anti-cytokine therapies that neutralize specific pro-inflammatory mediators, IPS represents a paradigm shift. It selectively preserves inflammation during the critical “resorption window” by avoiding potent anti-inflammatory agents in the acute phase, thereby harnessing endogenous repair mechanisms. This perspective explores three dimensions—molecular mechanisms, clinical evidence, and translational value—to reconcile inflammation’s dual nature. By deconstructing this “double-edged sword,” we aim to pioneer novel therapeutic approaches balancing pain control and tissue healing, ultimately advancing from symptom palliation to disease-modifying therapy.

## The cellular and molecular network driving inflammation-mediated resorption.

### Neovascular ingrowth: the “conduit engineering” of resorption

As an intrinsically avascular tissue, the intervertebral disc degeneration (IVD) establishes contact with the epidural vascular plexus upon herniation, triggering a precisely regulated “conduit engineering” process manifesting as a pathological angiogenesis network. Central to this process is the hypoxia-induced microenvironment, which stimulates nucleus pulposus cells (NPCs) to secrete vascular endothelial growth factor (VEGF). VEGF binds to the endothelial cell-specific receptor VEGFR2, activating downstream signaling pathways that drive the directed migration of vascular endothelial cells towards the herniated site, culminating in the formation of a functional neovascular network (19). Within this cascade, M2 macrophages and VEGF form a bidirectional regulatory loop: the anti-inflammatory cytokine IL-10 secreted by M2 macrophages further upregulates VEGF expression, reinforcing a positive feedback loop for angiogenesis (20). Conversely, the anti-angiogenic isoform VEGF_165_b antagonizes M2 polarization by inhibiting the S100A8/S100A9 signaling axis, constituting a negative feedback mechanism (21). Clinically, the newly formed vascular network (neovascularization) serves as a physical conduit for macrophage infiltration (22) while simultaneously transporting MMP precursors to the lesion site, facilitating extracellular matrix (ECM) degradation and tissue resorption (23). This cascade of angiogenesis-immune modulation-matrix degradation represents the critical pathological pathway from vascular invasion to tissue remodeling following disc herniation.

### Macrophage polarization: dynamic equilibrium regulating inflammation and repair

Macrophage polarization (M1/M2 switching) dynamically balances inflammation and repair, serving as a central hub for tissue homeostasis. During the inflammatory activation phase, pro-inflammatory cytokines such as TNF-α and IL-1β drive M1 polarization. Activated M1 macrophages, via the NF-κB signaling pathway, induce the expression of matrix metalloproteinases MMP-3 and MMP-9, which directly degrade the collagen network of the ECM (24, 25). Concurrently, M1 macrophages secrete IL-1β and TNF-α, activating apoptotic signaling pathways in NPCs (26, 27). Specifically, IL-1β induces mitochondrial-dependent apoptosis (e.g., via caspase cascade activation) and suppresses the synthesis of ECM components like collagen type II, leading to the loss of structural integrity in the IVD and ultimately accelerating the degenerative process (28).

Counterbalancing this is the M2 anti-inflammatory/repair phenotype. M2 macrophages secrete cytokines like IL-10 and TGF-β to establish an immunosuppressive microenvironment and upregulate MMP expression to promote cellular debris clearance (29). Notably, M2 macrophages transmit the HIF-1α/VEGF signaling axis via exosomes, directly stimulating angiogenesis and tissue regeneration (30). This phenotypic switching is finely regulated by multiple mechanisms: autophagy flux enhancers (e.g., theaflavin-3,3’-digallate) can drive M2 polarization by promoting lysosomal degradation pathways, demonstrating therapeutic potential in collagen-induced arthritis models (25). At the epigenetic level, the DNA methyltransferase inhibitor 5-Aza upregulates the M2 marker arginase-1 (Arg-1) while inhibiting the release of the pro-fibrotic factor TGF-β1, revealing the critical role of epigenetic modifications in determining polarization direction (31). This spatiotemporal switching between M1 and M2 phenotypes constitutes a comprehensive regulatory network governing the transition from inflammatory clearance to repair and reconstruction following tissue injury.

### Cell death and matrix remodeling: synergistic action of the apoptosis-autophagy axis

Within the resorption process of herniated IVDs, cell death and matrix remodeling form a precisely coordinated network via the apoptosis-autophagy axis. Apoptosis serves as a core pathway for clearing redundant cells, driven by the TNF-α/caspase-3 signaling axis to execute programmed cell death in NPCs, releasing apoptotic bodies containing damage-associated molecular patterns (DAMPs) (32). Notably, M2 macrophages secrete IL-10, creating a protective microenvironment. This directly inhibits caspase-3 activity and reduces apoptosis in adjacent cells—a key negative feedback mechanism (33). Simultaneously, the autophagy system initiates cytoplasmic component turnover through upregulated LC3-II protein expression, efficiently clearing damaged organelles and misfolded proteins via the lysosomal pathway, thereby acting as a “scavenger” in cellular homeostasis maintenance. Studies indicate (34) that deficiency in the Nrf2 transcription factor significantly suppresses autophagic flux, leading to the accumulation of undegraded protein aggregates. This exacerbates M1 macrophage polarization via the ROS-NF-κB axis, forming a vicious cycle contributing to pathological changes like lung injury. Regarding matrix remodeling, the dynamic balance between MMP-3/9 and TIMP-1 determines the degradation threshold of the collagen network: pathological disintegration of collagen fibers occurs when MMPs are excessively activated or TIMP-1 expression is insufficient. Furthermore, reactive oxygen species (ROS) accumulation can directly inhibit TIMP-1 enzymatic activity through oxidative modification, exacerbating matrix degradation imbalance (35). Notably, antioxidants like cerium-manganese nanozymes, by scavenging excess ROS, can effectively restore the MMP/TIMP enzymatic activity ratio, offering novel strategies for matrix homeostasis reconstruction. This three-dimensional regulatory network of apoptosis-autophagy-matrix degradation constitutes the core mechanism for cellular component renewal and tissue structural remodeling during herniated disc resorption. The resorption cascade initiates with hypoxia-induced VEGF secretion, which recruits vascular endothelial cells to form neovessels (Days 1–7). These neovessels enable macrophage influx, where early M1 activity (Days 3–14) degrades ECM components, followed by M2 polarization promoting repair. Concurrently, TNF-α/caspase-3-mediated apoptosis peaks (Days 7–28), releasing DAMPs that amplify M2 activation while autophagy clears ROS/protein aggregates to restore MMP/TIMP balance.

**Figure 1:**
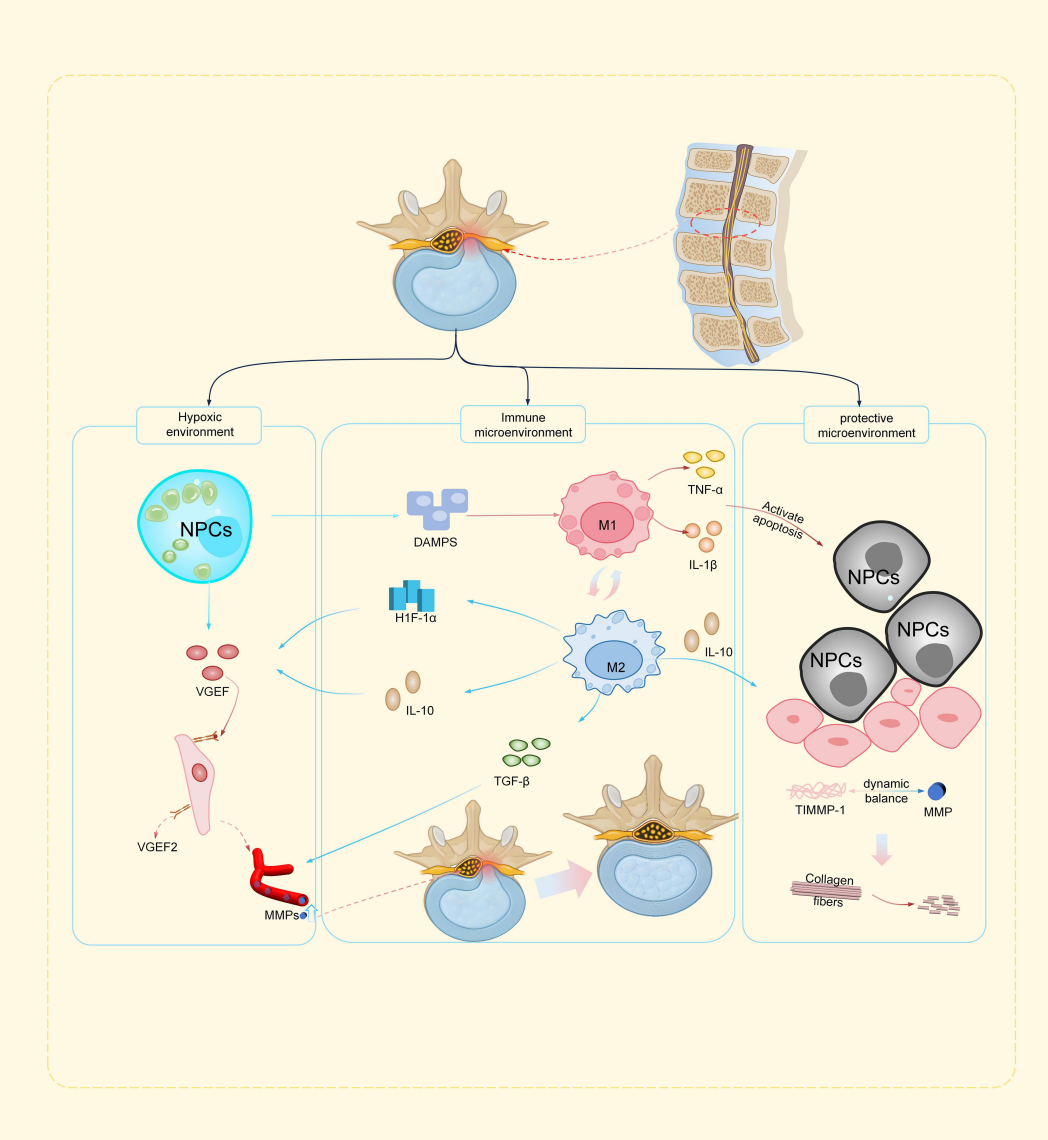
Schematic illustration of the inflammatory cascade driving lumbar disc herniation resorption **(a)** Hypoxic environment: Hypoxia-induced VEGF secretion by nucleus pulposus cells initiates neovascularization, forming vascular conduits for macrophage infiltration and MMP delivery. **(b)** Immune microenvironment: M1 macrophages drive ECM degradation via TNF-α/MMPs, while M2 macrophages promote repair through (IL-10/TGF-β/HIF-1α signaling, with epigenetic/autophagy regulators fine-tuning polarization. **(c)** Protective microenvironment: Apoptosis (TNF-α/caspase-3) releases DAMPs, counterbalanced by M2-derived IL-10 and autophagy-mediated debris clearance. ROS/MMP-driven matrix degradation is mitigated by antioxidants, restoring ECM homeostasis.

**Figure 1** illustrates the tripartite inflammatory cascade governing lumbar disc herniation resorption, integrating neovascular conduit engineering, dynamic macrophage polarization (M1/M2 balance), and the apoptosis-autophagy-matrix remodeling axis, which collectively mediate the transition from inflammatory injury to reparative tissue homeostasis.

The inflammatory response in spontaneous resorption is not merely pathological destruction but a precisely programmed tissue-repair mechanism. Its beneficial effects are achieved through multi-layered immunoregulation: firstly, as core executors, macrophages perceive DAMPs released from herniated nucleus pulposus via pattern recognition receptors (PRRs), triggering secretion of pro-inflammatory factors (TNF-α, IL-1β) to initiate immune cascades (36, 37). This process eliminates necrotic tissue fragments through lysosomal pathways while establishing a reparative microenvironment via the angiogenesis-matrix remodeling axis (38). The neovascular system provides physical channels for macrophage migration and delivers matrix metalloproteinases (MMPs) to lesion sites, with MMP-3/9 degrading collagen networks to enable tissue restructuring (39). Notably, caspase-1-mediated proteolytic cleavage of gasdermin D (GSDMD) by activated inflammasomes represents the critical step initiating pyroptosis (40), while pyroptosis-released inflammatory factors may indirectly promote VEGF expression, forming an “inflammation-angiogenesis-matrix degradation” positive regulatory circuit (41). Although specific cellular infiltration density and quantification efficiency remain unclarified, this dynamic equilibrium mechanism likely constitutes the core pathophysiological framework for LDH resorption. While the proposed temporal sequence of inflammatory resorption phases is mechanistically plausible, future studies using longitudinal imaging or serial biomarker assays are needed to validate these dynamics *in vivo*.

## The dual role of inflammation and the therapeutic rationale for inflammation preservation strategies: risks and regulatory approaches

The inflammatory response in spontaneous resorption of LDH is not merely pathological destruction but a precisely programmed response essential for tissue repair. Inflammation in LDH exhibits a dualistic nature: it is a necessary driver for resorption, yet inappropriate intervention can suppress this process. This duality forms the core logic of inflammation preservation strategies—retaining inflammation to facilitate “self-healing.

### The anti-inflammatory treatment paradox: comprehensive suppression disrupts physiological repair

Conventional anti-inflammatory drugs are widely used to alleviate inflammation-related symptoms, such as pain and swelling. However, their core mechanism of blocking the inflammatory cascade creates a paradox: while mitigating pathological states, global suppression may potentially interfere with physiological repair processes. Specifically, this “non-selective suppression” characteristic may antagonize the natural regulatory processes required for tissue resorption or healing, particularly evident with NSAIDs.

NSAIDs, including traditional non-selective agents and selective COX-2 inhibitors, primarily act by competitively inhibiting cyclooxygenase (COX) to block prostaglandin (PG) synthesis (42). COX enzymes play a central role in inflammatory pathways, mediating the biotransformation of arachidonic acid to generate various PGs. While most studies report reduced disc resorption rates with NSAID use, certain clinical scenarios—such as short-term (<7 days) administration for non-sequestered herniations—show neutral effects on resorption. This may reflect preserved VEGF signaling due to limited intervention depth and maintained neovascularization potential. These PGs are not only biochemical mediators of inflammation and pain but are also theoretically involved in angiogenesis and tissue repair. However, global suppression of the COX pathway may indiscriminately inhibit beneficial signals, leading to disruption of the “inflammation-repair” balance.

### Precision intervention pathways for inflammation preservation strategies

The core of inflammation preservation strategies lies in implementing targeted interventions that block excessive damage while preserving the reparative functions of inflammation. Any carrier system employed requires rigorous biocompatibility assessment to avoid foreign body reactions inducing secondary inflammation. Research exploring spontaneous resolution mechanisms provides a foundation for these strategies. Zhao et al. (43) through a case study, confirmed spontaneous resorption of the nucleus pulposus in a patient with severe L5/S1 herniation after two years of conservative management (including NSAIDs, thermotherapy, and exercise therapy), accompanied by significant decreases in inflammatory markers and symptom improvement. The study indicated that spontaneous resolution involves macrophage-mediated phagocytosis and reductions in inflammatory cytokines (e.g., IL-6, TNF-α), providing theoretical support for conservative management. Similarly, a systematic review (44) analyzing predictors and mechanisms of nucleus pulposus (NP) spontaneous resorption demonstrated that approximately 50–70% of patients under conservative management (≥3 months) exhibited significant resorption, primarily attributed to neovascularization and immune cell infiltration reducing the inflammatory response. Clinical research further corroborates this (45): the resorption rate was lower in groups receiving early anti-inflammatory medication compared to those managed with inflammation preservation approaches. Consequently, avoiding anti-inflammatory drugs like NSAIDs resulted in a 100% resorption rate in acute LDH patients. Current evidence (46, 47) indicates that NSAIDs may exert inhibitory effects on bone healing. However, the distinct pharmacological profiles between COX-2 selective inhibitors and non-selective NSAIDs, along with the emergence of novel compounds, present opportunities to develop more nuanced and patient-specific treatment regimens for individuals undergoing IPS protocols.

Deeper regulatory mechanisms focus on modulating cell death modalities. Small-molecule inhibitors targeting the caspase-1/GSDMD complex structure can selectively suppress excessive pyroptotic damage while preserving the contribution of apoptosis/autophagy pathways to tissue repair (48).

During apoptosis, mitochondrial BAK protein, activated by BH3-only molecules, sequesters ATP within LC3-positive vesicles via a non-canonical autophagy pathway. This molecular sequestration mechanism reduces ATP efflux as a DAMP, thereby inhibiting phagocyte activation and the secretion of pro-inflammatory cytokines like IL-1β (49). This finding mechanistically aligns with (IVD research: IL-1β accelerates IVD via NLRP3 inflammasome-mediated pyroptosis, while activating the nuclear receptor NR1D1 suppresses this pathway. The NR1D1 agonist SR9009 effectively mitigates inflammatory damage and promotes extracellular matrix synthesis by regulating the NR1D1/NLRP3/IL-1β axis, offering a novel therapeutic target for IVD (50). This multi-dimensional regulatory strategy provides a paradigm shift from “inflammation suppression” to “inflammation remodeling” for LDH treatment (51).

### Translational applications of inflammation preservation strategies

The transition of inflammation preservation strategies from theory to clinical application is underway, centered on precise modulation rather than comprehensive suppression of inflammation. Conservative management remains the first-line approach for LDH, with current clinical pathways encompassing three main directions: pharmacotherapy, physical therapy, and regenerative medicine, all aiming to control inflammation and promote repair.

Multiple sources address this theme: A 2025 review (52) summarized recent non-surgical strategies. Platelet-rich plasma (PRP) and bone marrow aspirate concentrate (BMAC) modulate macrophage polarization from pro-inflammatory M1 to anti-inflammatory M2 phenotypes, inhibiting the release of inflammatory cytokines like IL-1β and TNF-α, thereby slowing disc degeneration. Based on World Federation of Neurosurgical Societies (WFNS) Spine Committee recommendations, Yaman et al. (53) stated that conservative treatment (including NSAIDs, thermotherapy, and exercise) is effective in ≥70% of patients with mild-to-moderate LDH, achieving symptom relief by reducing inflammatory cytokine levels and oxidative stress.

Systemic administration of traditional NSAIDs, due to their non-selective inhibition of PG synthesis, may concurrently block MMP-mediated enzymatic clearance of herniated material. Novel epidural/selective nerve root blockade techniques enable precise, image-guided drug delivery to the inflammatory site. These techniques deliver anti-TNF-α antibodies directly to areas of nerve root compression, targeting pro-inflammatory factors (e.g., TNF-α, IL-6, PGE2) released by herniated NP material (54). A meta-analysis of randomized controlled trials (55) demonstrated that tailored exercise protocols for LDH (e.g., core muscle training) downregulate pro-inflammatory factors while upregulating anti-inflammatory mediators, improving functional scores without completely blocking inflammatory pathways. This evidence highlights the translational potential of inflammation preservation strategies in non-pharmacological treatments.

**Regenerative medicine strategies are particularly promising.** Researchers developed a dual-network bio-sealant loaded with extracellular vesicles for immunomodulation and annulus fibrosus (AF) repair (56). By downregulating inflammatory cytokines (e.g., TNF-α, IL-1β) and activating anti-inflammatory pathways (e.g., eNOS/VEGFa), it significantly reduced inflammatory infiltration in the herniation zone, with tissue regeneration and functional recovery observed in animal models after 4 weeks. Furthermore, hydrogels demonstrate biocompatibility and biodegradability matching neuronal tissues (57). Studies show that hydrogel scaffolds loaded with the anti-inflammatory molecule TGF-β1 can neutralize pro-inflammatory factors in the microenvironment, inhibit MMP-3/13 expression, and protect the extracellular matrix (58). Utilizing hydrogels to deliver anti-inflammatory cytokines (e.g., IL-4, TGF-β1) or stem cells promotes the shift from M1 (pro-inflammatory) to M2 (reparative) phenotypes (59). Concurrently, Yu et al. (60) employed menstrual blood-derived mesenchymal stem cells (MenSCs) combined with collagen I gel in a post-discectomy rat model. They confirmed that stem cells secrete the anti-inflammatory factor IL-4, promoting disc tissue remodeling, reducing inflammatory damage, and improving biomechanical stability. A systematic review (61) showed that percutaneous endoscopic lumbar discectomy (PELD) combined with platelet-rich plasma (PRP) injection significantly reduces postoperative recurrence rates in LDH. PRP suppresses local inflammation by releasing anti-inflammatory factors (e.g., IL-1Ra) and promotes disc tissue repair.

## Future directions: precision-targeting of inflammation preservation strategies and clinical translation pathways

The Inflammation Preservation Strategy (IPS) represents a paradigm shift in lumbar disc herniation (LDH) management. However, its clinical translation faces critical challenges. Future research must focus on the following core directions to advance this strategy from concept to precision practice:

### Direction 1: clinical translation of dynamic inflammation phenotyping technologies

Establishing a non-invasive, dynamic monitoring system for inflammatory phenotypes in LDH is a pivotal technological breakthrough for the precise implementation of IPS. A multi-scale inflammation assessment framework, integrating molecular biomarkers with macro-imaging features, can be achieved through the deep integration of multi-modal radiomics and liquid biopsy technologies.

ROS-responsive nanosensors enable real-time capture of local inflammatory signal changes within the microenvironment. PET-CT can detect specific inflammatory targets. Additionally, microsampling techniques (e.g., capillary microsampling, dried blood spots) facilitate continuous monitoring of inflammatory biomarkers (62). Zhao et al. (63) designed a DNA nano-orchestrator exhibiting ROS-responsive component release. *In vitro*, it is efficiently internalized by cells, stimulates Toll-like receptor 9 (TLR9) in dendritic cells (DCs), inhibits autophagy, and enhances major histocompatibility complex class I (MHC-I) expression. It also activates systemic adaptive immunity by increasing the infiltration of DCs and CD8+ T cells. Li et al. (64) further propose that the relationship between paraspinal muscle properties and bone mineral density, assessed via MRI and quantitative computed tomography (QCT), can extend this multi-modal imaging approach to inflammatory phenotype analysis.

### Direction 2: functional iteration of intelligent biomaterials

Traditional discectomy can trigger postoperative inflammatory cascades due to mechanical trauma, potentially leading to secondary annular rupture in residual nucleus pulposus tissue, exacerbated inflammation, and reherniation risk. Studies indicate that unaddressed annular defects post-discectomy increase reherniation rates, while biomaterials can locally deliver anti-inflammatory factors and provide repair scaffolds (65). Consequently, developing microenvironment-responsive scaffold materials capable of dynamically sensing local inflammation levels and regulating the inflammation-repair balance is crucial (66).

Recent research (67) reports a multifunctional dual-network (DN) hydrogel composed of a physically cross-linked carboxymethyl chitosan (CMCS) and tannic acid (TA) network, combined with a chemically cross-linked acrylamide (AM) network. This hydrogel integrates high strength, adhesion, biocompatibility, and anti-inflammatory properties. Treatment significantly reduces levels of inflammatory cytokines during IVD and partially restores disc biomechanics. Additional research avenues include self-powered triboelectric-responsive microneedle devices integrating targeted optogenetically engineered extracellular vesicles for controlled release, aiming to restore functional homeostasis in aged nucleus pulposus cells and promote precision repair of inflammatory disc degeneration (68). Future research should focus on deep modulation of the material-immune interface, integrating extracellular matrix (ECM)-mimetic ligands with immune checkpoint modulators to engineer clinical biomaterials with combined immune-evasive and inflammation-reprogramming functions, creating an immunologically favorable microenvironment for endogenous disc repair.

### Direction 3: evidence-based advancement of clinical translation pathways

Despite the diversified development of clinical intervention strategies for LDH, evidence-based research on inflammation-retaining protocols still faces the fundamental challenge of lacking standardized pathways. Substantial controversies persist in current treatment paradigms regarding surgical indications, conservative protocol selection, and complication prevention, leading to the coexistence of overtreatment and undertreatment (69). To address this impasse, future research could establish a clinical translation framework integrating phenotypic precision stratification, individualized therapeutic decision-making, and dynamic prognostic assessment. This approach would enable comprehensive precision management from diagnostic classification to treatment planning through systematic integration of disease heterogeneity markers and therapeutic response biomarkers. The critical window for IPS intervention is defined by the temporal dynamics of inflammation and tissue remodeling. During this interval, inflammatory processes are maximally active, creating an optimal environment for nucleus pulposus resorption through angiogenesis and pro-repair macrophage activity (70). Premature or delayed administration of anti-inflammatory agents during this window may disrupt the delicate balance between catabolic inflammation and anabolic repair, potentially reducing resorption efficacy.

In the context of staged disease intervention, patients in acute inflammatory phases may benefit from minimally invasive transforaminal endoscopic decompression combined with drug-eluting anti-inflammatory scaffold implantation, achieving localized inflammatory microenvironment modulation via controlled glucocorticoid release. During subsequent tissue repair phases, bioactive scaffolds loaded with mesenchymal stem cell-derived exosomes containing miR-21-5p and TGF-β1 regulatory factors could be introduced to promote disc matrix synthesis and neural regeneration (71). Notably, the postoperative management module incorporates spinal surgery experience (72) by integrating the complication prediction system with longitudinal functional recovery data, facilitating proactive monitoring and prevention of adverse events and surgical sequelae. As underscored in recent studies, “Addressing these factors – regulatory compliance, scalable production, cost-effectiveness, and rigorous safety assessments – is crucial for advancing biomaterials from the lab to clinical applications” (73). Clinician-scientist partnerships are critical to overcoming translational barriers. Looking ahead, “Collaboration between clinicians and scientists holds the key to revolutionizing patient care through biomaterial science” (74).

### Temporal prioritization of research goals

To operationalize the ambitious future directions, we propose a tiered implementation framework: short-term (1–3 years) efforts will validate dynamic inflammation phenotyping through multi-center radiomics studies, medium-term (3–5 years) initiatives will advance intelligent biomaterial iteration via FDA-regulated hydrogel trials, and long-term (5–10 years) objectives will establish clinical translation pathways through international consortium-driven registries, aligning with NIH Stage Model principles for actionable research prioritization.

## Conclusion

The Inflammation Preservation Strategy represents a transformative innovation in LDH management. Inflammation is not only a key driver of radicular pain but also an indispensable physiological engine for the spontaneous resorption of herniated nucleus pulposus. This process relies on a precisely regulated cascade: neovascularization provides the “logistical conduit,” macrophage infiltration executes matrix degradation and debris clearance, and the synergistic action of the apoptosis-autophagy axis facilitates final cellular turnover and tissue remodeling.

Traditional pan-anti-inflammatory strategies centered on non-steroidal anti-inflammatory drugs (NSAIDs) and glucocorticoids, while effective for acute pain relief, employ non-selective global suppression. Both basic and clinical evidence confirms this significantly interferes with, or even blocks, this physiological repair process, leading to reduced resorption rates and increased recurrence risk. Supplementary Material 1 presents a hierarchical evidence matrix that categorizes key findings by research strata (basic/translational/clinical) and thematic dimensions (mechanistic pathways/biomaterial innovation/interventional efficacy).

The core logic of IPS is therefore revolutionary: During specific stages, particularly for sequestered/migrated LDH patients within the “inflammation-driven resorption window”(approximately Days 3–14 post-injury) in patients with sequestered/migrated lumbar disc herniation (LDH)—potent anti-inflammatory agents should be judiciously avoided or strictly limited. This phase, bridging the inflammatory (Days 0–4) and proliferative stages (Days 4–14) of tissue repair as delineated in contemporary wound healing frameworks, represents a critical period when inflammatory processes are maximally active yet precisely regulated. Instead, employ physical therapy (e.g., thermotherapy/exercise), spatiotemporally responsive biomaterials like hydrogels or nanocarriers, and repurposed traditional agents should be employed to achieve “precision modulation” rather than “comprehensive suppression” of the inflammatory microenvironment (75). The goal is to effectively manage pain thresholds while maximally preserving and optimizing the inflammation response’s capacity for herniated tissue clearance and repair, ultimately enabling a transition from “palliative symptom relief” to “disease-modifying therapy.

## Data Availability

The original contributions presented in the study are included in the article/**Supplementary Material**. Further inquiries can be directed to the corresponding authors.
